# Dimerization of Protegrin-1 in Different Environments

**DOI:** 10.3390/ijms11093177

**Published:** 2010-09-09

**Authors:** Victor Vivcharuk, Yiannis N. Kaznessis

**Affiliations:** Department of Chemical Engineering and Materials Science, University of Minnesota, Minneapolis, MN 55455-0132, USA

**Keywords:** protegrin, potential of mean force, dimerization

## Abstract

The dimerization of the cationic *β*-hairpin antimicrobial peptide protegrin-1 (PG1) is investigated in three different environments: water, the surface of a lipid bilayer membrane, and the core of the membrane. PG1 is known to kill bacteria by forming oligomeric membrane pores, which permeabilize the cells. PG1 dimers are found in two distinct, parallel and antiparallel, conformations, known as important intermediate structural units of the active pore oligomers. What is not clear is the sequence of events from PG1 monomers in solution to pores inside membranes. The step we focus on in this work is the dimerization of PG1. In particular, we are interested in determining where PG1 dimerization is most favorable. We use extensive molecular dynamics simulations to determine the potential of mean force as a function of distance between two PG1 monomers in the aqueous subphase, the surface of model lipid bilayers and the interior of these bilayers. We investigate the two known distinct modes of dimerization that result in either a parallel or an antiparallel *β*-sheet orientation. The model bilayer membranes are composed of anionic palmitoyl-oleoyl-phosphatidylglycerol (POPG) and palmitoyl-oleoyl-phosphatidylethanolamine (POPE) in a 1:3 ratio (POPG:POPE). We find the parallel PG1 dimer association to be more favorable than the antiparallel one in water and inside the membrane. However, we observe that the antiparallel PG1 *β*-sheet dimer conformation is somewhat more stable than the parallel dimer association at the surface of the membrane. We explore the role of hydrogen bonds and ionic bridges in peptide dimerization in the three environments. Detailed knowledge of how networks of ionic bridges and hydrogen bonds contribute to peptide stability is essential for the purpose of understanding the mechanism of action for membrane-active peptides as well as for designing peptides which can modulate membrane properties. The findings are suggestive of the dominant pathways leading from individual PG1 molecules in solution to functional pores in bacterial membranes.

## 1. Introduction

Protegrin-1 (PG1) is a potent antimicrobial, *β*-hairpin, cationic peptide [1,2]. A simple model that explains how PG1 kills bacteria involves oligomeric (typically octameric or decameric) peptide pores in anionic lipid bilayer membranes [3,4], which mimic the inner membrane of Gram-negative bacteria. Cytosolic potassium is released through these pores, and sodium enters the cell, causing a significant transmembrane potential decay, a subsequent cell volume expansion and lethal membrane rupture [5,6]. NMR experiments indicate that PG1 dimers are structural prerequisites of PG1 pores inside anionic lipid membranes and of PG1 *β*-sheets on the surface of cholesterol-containing zwitterionic lipid bilayers, which, in turn, mimic mammalian cell membranes [3,7].

Two distinct dimer packing modes are prevalently observed, depending on the studied environments: parallel and antiparallel. The parallel structure is in an NCCN packing mode, where N and C stand for the peptide’s N-terminus and C-terminus, respectively. In particular, parallel dimers have been observed on the surface of cholesterol-containing zwitterionic lipid bilayers, whereas antiparallel dimer structures have been observed on the surface of dodecylphosphocholine micelles. Importantly, parallel structures have been observed inside anionic lipid bilayers. Indeed, the model of the octameric pore determined by NMR [3] and simulated by molecular dynamics [4] comprises of four distinct, structurally stable, parallel dimers.

In order to develop mechanistic explanations of the antimicrobial activity of PG1, substantial efforts have been expended on determining how protegrin monomers and dimers interact with model membranes [4,8–16]. For example in [15] the energetics of protegrin binding and inserting in model membranes were determined. The preferred orientations and conformations of PG1 were also established in model membranes [16]. Less attention has been paid to the phenomenon of dimerization, which is notable in its own right, since PG1 carries a (+7) charge. Indeed, little is known regarding the thermodynamics and kinetics of peptide-peptide aggregation at the molecular level and how the environment dictates PG1 dimerization.

In this work, we attempt to address this gap by investigating the dimerization of PG1 in various environments. Understanding on a molecular basis how peptides of oligomers structures maintain stability remains an important challenge, since detailed knowledge of dimerization through calculation of free energy and monitoring ionic bridge and hydrogen bond networks increases our basic understanding not only of pepetide oligomer structure and function, but also of the origin and progression of peptide selectivity to different membranes. Furthermore, it may suggest rules for the rational design and engineering of antibiotic peptides. We employ atomistic simulations to calculate the potential of mean force of dimer formation. In particular, we determine the energetically preferred structures in water, on the surface of lipid bilayers and inside the hydrophobic core of lipid membranes. We investigate the effects of hydrogen bonds between the peptides and ionic bonds between peptides and ions, or peptides and lipids. Illustrating the mechanism of dimerization contributes to our efforts to further explain the molecular mechanism of antimicrobial activity.

In what follows, we describe the details of the computer simulations and the calculation of the potential of mean force. We apply a variant of constrained molecular dynamics (MD) simulations and the thermodynamic integration method to determine the potential of mean force, and calculate the equilibrium binding constant and related adsorption free energy. We then present and discuss the results in the context of earlier work. Based on our results, we also speculate on the dominant kinetic pathways that PG1 follows from solution to pores.

## 2. Methods

### 2.1. Microscopic Models for PG1 Dimers in the Water Phase, on the Surface of a POPG:POPE Membrane and inside a POPG:POPE Membrane

**Figure 1:**
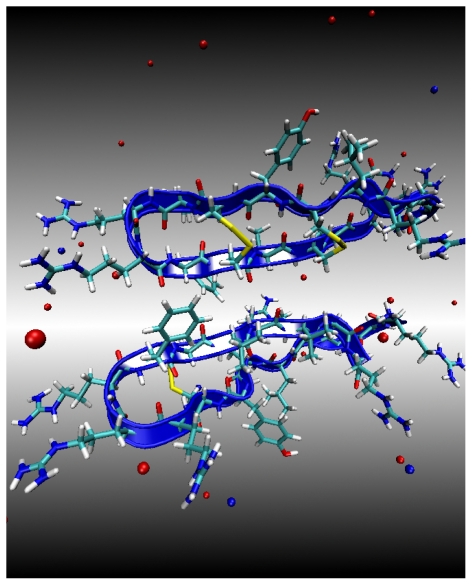
Structure of the PG1 dimer in the parallel *β*-sheet arrangement, in an NCCN packing mode. The peptide backbones are shown as yellow ribbons. The solution Na^+^ and Cl^−^ counterions are shown as red and blue spheres, respectively. The peptide residues Arg and Cys are also shown as sticks. Water molecules have been removed for clarity.

PG1 is an 18-residue cationic *β*-hairpin antimicrobial peptide (RGGRL CYCRR RFCVC VGR-NH2) [1]. PG1 dimerizes either in a parallel structure, hereafter denoted by PG1*_p_^d^*, or an antiparallel one, hereafter denoted by PG1*_a_^d^*. In Figure 1 the parallel dimer structure is illustrated. The structure of PG1*_p_^d^* has been determined by NMR [3]. The atomic coordinates were downloaded from the protein data bank (PDB code 1ZY6). Currently there is no atomistic resolution structure of the antiparallel structure. We constructed the initial antiparallel NCCN *β*-sheet arrangement (PG1*_a_^d^*) from the parallel configuration by rotating one PG1 peptide 180*°* about the Cys_15_ *α*-carbon atom to satisfy the antiparallel NCCN packing model for the PG-1 dimer, which was implied by the rotational-echo double-resonance solid state NMR [7]. The formation of PG1*_p_^d^* and PG1*_a_^d^* is investigated in the following three distinct environments:

#### Environment 1: Water Subphase

We simulated the formation of PG1*_p_^d^* and PG1*_a_^d^* in bulk water. We solvated the two peptides with nearly 7030 TIP3P water molecules, 34 chlorine ions, and 20 sodium ions. Chlorine and sodium ions were added to create 0.15 M physiological salt solution and to neutralize the charge of the two identical PG1 peptides.

#### Environment 2: Lipid Bilayer Surface

The PG1 dimers in the parallel PG1*_p_^d^* and antiparallel PG1*_a_^d^* *β*-sheet arrangements were placed on the surface of a mixed lipid bilayer, consisting of 224 lipids, including 56 anionic palmitoyl-oleoyl-phosphatidylglycerol (POPG) and 168 palmitoyl-oleoyl-phosphatidylethanolamine (POPE) in a 1:3 ratio (POPG:POPE). This is a composition previously used to model the inner membrane of Gram-negative bacteria [4]. Both PG1*_p_^d^* and PG1*_a_^d^* dimers were oriented parallel to the membrane such that one of the PG1 peptide backbones was parallel to the membrane along the *y* direction, with residues Cys6, Cys8, and Cys15 laying on the *xy*-plane. The center of mass of the peptides was positioned 29 *Å* from the center of mass of the membrane. This separation distance corresponds to a minimum free energy profile of the interaction between a dimer of protegin-1 in the parallel arrangement and a model lipid membrane [15]. Here we made the assumption that the free energy minimum for dimers in the parallel and antiparallel packing models is attained at the same distance from the membrane. The system is solvated with nearly 10800 TIP3P water molecules, 44 chlorine ions, and 86 sodium ions. Chlorine and sodium ions are added to create 0.15 M physiological salt solution and to neutralize the charge of the peptides and POPG head groups.

#### Environment 3: Lipid Bilayer Core

**Figure 2:**
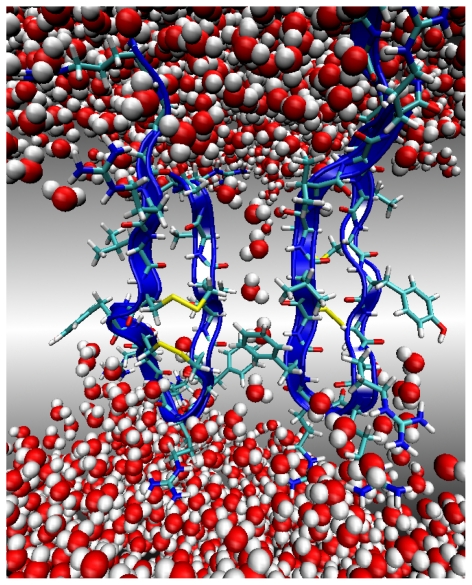
A snapshot at the end of a 4 ns simulation of the PG1 dimer in the parallel *β*-sheet arrangement inside the membrane. Peptides are shown in blue NewCartoon representation. Water is shown as van der Waals spheres. The solution Na^+^ and Cl^−^ counterions are shown as small yellow and large green spheres, respectively. The peptide Cys and Arg residues are shown as sticks. The sidechain atoms of the other residues and the bilayer lipid atoms are omitted for clarity.

To build a transmembrane complex for molecular dynamics (MD) simulations of dimers in the NCCN *β*-sheet PG1*_p_^d^* and PG1*_a_^d^* arrangements, we used the CHARMM-GUI membrane builder with the replacement method [17]. The peptide dimer was placed with its principal axis parallel to the bilayer normal and the dimer center of mass was located at the bilayer center of mass (Figure 2). For both dimer configurations, we use the solvated lipid bilayer system of 152 lipids (*i.e.*, 76 lipids in each leaflet) containing 114 POPE lipids and 38 POPG lipids. The system is solvated with nearly 4300 TIP3P water molecules with 29 chlorine ions, and 53 sodium ions. Chlorine and sodium ions are added to create 0.15 M physiological salt solution, to neutralize the charge of the peptides and POPG head groups. The surface area occupied by two peptides and used in the membrane builder is about 320 *Å*^2^ [12].

### 2.2. Molecular Dynamics Protocol

Our goal is to use molecular dynamics simulations to calculate a potential of mean force (PMF) for the dimerization of PG1. In the next section, we present the details of the PMF calculation. In this section we discuss the molecular dynamics simulation protocol. For each one of the three environments, and for each of two dimer conformations, a set of 9 different simulations is conducted with the center of mass of the two peptides at different distances. All systems were constructed in a rectangular volume cell using the program CHARMM [18] and CHARMM-GUI Modeler [17]. The CHARMM-27 force field [19] with CMAP corrections [20] was employed. All structures of the *β*-hairpin PG1 were generated with two disulfide bonds, amidated C-termini, and six positevly charged arginines reflecting the typical protonation state of arginine. An assumption that may be of limited accuracy is that the protonation state of PG1 does not change when PG1 is embedded inside the lipid bilayer. We should note though that the two ends of the PG1 structure, *i.e.*, the N- and C- termini on the one end and the *β*-hairpin on the other end, are both outside the lipid hydrophobic core and in contact with lipid headgroups. We used the NAMD software package [21] employing the Nose-Hoover-Langevin pressure controller [22,23] for all simulations. The pressure was set to 1 atmosphere with a piston period set to 200 fs and piston decay of 100 fs. The system was heated to 310 K (above the gel-liquid crystal phase transition of the mixed membrane [24] ) in increments of 30 K, running for 5000 steps at each temperature. After minimization and heating, all simulation boxes were equilibrated for 4 ns in the NPT ensemble. The water molecules were simulated using the TIP3P water model [25]. The van der Waals interactions were smoothly switched off over a distance of 4 *Å*, between 8 and 12 *Å*. The electrostatic interactions were simulated using the particle mesh Ewald summation with a grid of approximately 1 point per 1 *Å* apart in each direction [26]. During equilibration, in all simulation boxes for Environments 2 and 3, the area per lipid remained constant at the mixed (1:3) POPG:POPE system average at a value, 63.7 ± 1.4 *Å*^2^, and 63.2 ± 1.7 *Å*^2^, respectively. The average dimensions of the equilibrated simulation box for Systems 1, 2 and 3 are 67.3 × 65.6 × 53.2 *Å*, 84.1 × 84.8 × 82.7 *Å* and 63.4 × 80.8 × 62.2 *Å*., respectively.

### 2.3. Construction of Potential of Mean Force (PMF) for the Formation of PG1_p_^d^ and PG1_a_^d^

We calculate the potential of mean force, *W*(*D*), along a single reaction coordinate corresponding to the separation distance, *D*, between the centers of mass of two peptides. The separation intervals include the distance between the center of mass of the two peptides for a stable dimer structure as determined by NMR experiments [3] in a POPC membrane. A simple geometry is implemented to represent the two peptides: we consider each PG1 peptide as a cylinder of radius *a* and length *L*. The two cylinders are always parallel to each other along their long axis. For all systems the *y*-coordinate is defined by the vector connecting the centers of mass of the two peptides. The principal axis of both peptides is parallel to the *x*-axis. In Environment 2, the two cylinders lie parallel to the membrane surface and for Environment 3 both cylinders lie with their long axes perpendicular to the membrane surface. We restrict ourselves to the two orientational modes, PG1*_p_^d^* and PG1*_a_^d^*, in which the peptide backbones remain parallel to each other in an NCCN packing mode as observed in [3,7]. We simplify the analysis representing PG1 by a cylinder with an “effective” radius *a* = 4.5 *Å*. Thus, the minimum separation distance between two peptides is chosen as 2*a* = 9 *Å*. The simulation procedure is broken down into several stages:

- The two peptides are positioned in either the parallel or antiparallel orientation, at a distance *D*. The peptide separation, *D*, ranges from 9 *Å* to 25 *Å* in increments of 2 *Å*. There are thus 54 systems constructed (two orientations, three environments, nine separation distances). We should note that for PG1*_p_^d^* in Environment 3, we added another separation distance of *D* = 27 *Å*, in order to ascertain that the PMF attains a plateau at long distances, as discussed in more detail in Section 3. Each of the 55 constructed system is then equilibrated over 4 ns in the NPT ensemble. During this equilibration the PG1 peptides are restrained using harmonic springs with a a force constant 20 (kcal/mol)/*Å*^2^ applied to all peptide backbone atoms.
- A 4 ns production run is then conducted for each of the 55 initial equilibrated systems. In production runs, and in order to restrain the peptides and their orientations, we use harmonic springs coupled to the three carbon *C_B_* backbone atoms of Arg1, Arg10 and Cys15. All spring constants were 20 (kcal/mol)/*Å*^2^. In addition, and in order to better ascertain convergence of the PMF calculation, we extended the simulation of the parallel configuration, PG1*_p_^d^*, inside the membrane by an additional 4 ns, to 8 ns, for all examined distances.
- The instantaneous restraint forces are computed for each of the 55 system configurations for PG1 dimers with a sampling interval of 0.2 ps, and averaged to obtain the mean force *F̄* (*D*) = −*F̄^res^* (*D*) for each position, where *F̄^res^* (*D*) is the force exerted on the harmonic restraint springs. We concentrate our efforts on reducing the statistical errors. A difficulty is that, on short time scales, the results are highly correlated, and thus unsuitable for statistical analysis. We find that the correlation time for estimating the error due to solvent force fluctuations is about 0.1 ns, and membrane fluctuations and systematic error due to the harmonic restraints require data for no less than 0.5 ns to compute reliable average forces. Using the block-averaging method [27] we find the statistical errors in *F̄^res^* (*D*) to be within 0.4 (kcal/mol)/*Å* in all cases. The total sampling time must therefore be long enough to ensure a collection of uncorrelated configurations.
- The PMF can be evaluated by applying the mean force integration method which was developed for the PMF calculation of a peptide in the vicinity of a neutral POPC membrane [28]. This method is a variant of constrained MD and thermodynamic integration [29–34]. In particular, the PMF, *W*(*D*), is calculated using (1), where the integration over the *D* coordinate is performed using the trapezoidal rule:

(1) $$W(D)=-\int_{\infty }^{D}\bar{F}(D^{\prime })dD^{\prime }$$

An estimate of the relative equilibrium binding constant between the two PG1 peptides is

(2) $$K=\frac{1}{D_{max}-D_{min}}\int_{D_{min}}^{D_{max}}dD{\text{e}}^{-\beta W(D)}\equiv {\langle {\text{e}}^{-\beta W}\rangle }_{\Delta l}$$

where *β* = 1/*k_B_T* with *k_B_* Boltzmann’s constant, *D_min_* is the minimum separation distance or collision radius between the two peptides and *D_max_* determines the radius of binding or separation distance which divides the free and bound volumes [28]. The relative dimerization free energy Δ*G*^0^ is obtained via the following expression

(3) $$\Delta G^{0}=-k_{B}T\text{ln}K$$

In Section 3, we present and analyze the MD results for our systems with the help of Equations (1) and (3).

## 3. Results and Discussion

### 3.1. Binding Affinity of PG1 Peptides in the Parallel and Antiparallel β-Sheet Arrangements

**Figure 3:**
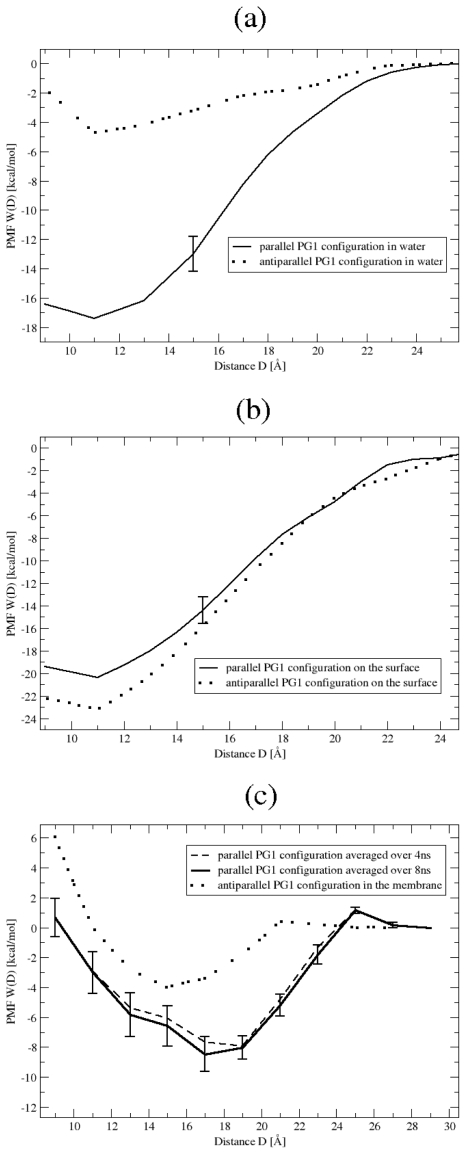
The potential of mean force, W(D), as a function of the distance between two peptides for the PG1 dimer in parallel (solid line) and antiparallel (dottet line) *β*-sheet arrangements in **(a)** water, **(b)** on the membrane surface. Each data point for W(D) represents the mean of eight 0.5 ns simulations. The PMF for the parallel and antiparallel arrangements inside the membrane **(c)** are also shown. For untiparallel structure (dotted line) each data point for W(D) represents the mean of eight 0.5 ns simulations. For parralel structure we have two plots. The dashed line represents the mean of eight 0.5 ns simulations, whereas solid line represents the mean of sixteen 0.5 ns simulations.

The potential of mean force, *W*(*D*) for protegrin dimerization is calculated for six systems. Two distinct protegrin dimer structures were examined each in three separate environments. In Figure 3 the six PMFs are plotted as a function of the peptide-peptide distance, *D*. For parallel and the antiparallel orientations in Environments 1 and 2, as well as for the antiparallel configuration in Environment 3, each value of *W*(*D*) represents the mean of eight 0.5 ns simulations, and the error bar represents the standard deviation. For the parallel configuration PG1*_p_^d^* inside the membrane (Figure 3 (c)) we present two plots. The dashed line represents the mean of the first eight 0.5 ns simulations. The solid line represents the mean of the first sixteen 0.5 ns simulations. As observed, the PMF values remain constant within the standard deviations. It is also observed that the PMF reaches a zero energy plateau at large D.

We find that for bulk water, the PMF minimum is about −17.5 kcal/mol for the PG1*_p_^d^* complex at a separation distance *D* = 11 *Å*. The PMF minimum is −4.8 kcal/mol for PG1*_a_^d^* at *D* = 11 *Å*. We can then rather confidently remark that the protegrins in parallel form a more stable dimer in water than in an antiparallel configuration. The calculated PMF has a minimum in a position which corresponds to equilibrium positions of the two PG1 peptides in the parallel dimer PG1 structure determined by NMR experiments [3]. On a bilayer surface, the PMF minimum is −20.4 kcal/mol for the PG1*_p_^d^* complex at a separation distance of *D* = 11 *Å*. The PMF minimum is −23.2 kcal/mol for the PG1*_a_^d^* at the same distance *D* = 11 *Å*. This result points to a stabler PG1*_a_^d^* dimer on the POPE/POPG surface, although the difference between the PMFs of the two dimer configurations is not pronounced enough to draw definite conclusions. Finally, when the peptides are inserted in parallel inside the POPE/POPG membrane, the PMF exhibits a rather broad minimum plateau which extends from approximately *D* = 17 *Å* to *D* = 19 *Å* and which is about −8.0 kcal/mol. The minimum is −3.8 kcal/mol for the PG1*_a_^d^* at *D* = 15 *Å*. Thus, the PG1*_p_^d^* dimer in the transmembrane configuration forms a relatively stronger binding complex compared to PG1*_a_^d^*.

From these results we can see that the separation peptide distances corresponding to the PMF minimum for Environments 1 and 2 are approximately equal to the distance of 11 *Å* obtained from NMR experiment for the parallel dimer inside a POPC membrane. The results of MD simulations for PG1 dimers inserted inside a POPE:POPG membrane apparently deviate from the NMR measurements, which were conducted for a zwitterionic POPC membrane. Clearly the type of phospholipids significantly impacts the interaction between PG1 peptides. It would be interesting to use simulations to investigate PG1 dimerization in zwitterionic lipid membranes, but such calculations are beyond the scope of the present study. Notably, the parallel dimer configuration appears to be overall more favorable than the antiparallel one. Remarkably, dimerization appears to be more favored on the surface of a lipid bilayer than either in the solvent or inside the membrane. In earlier work [15], we determined that protegrin monomers are more likely found on the surface of POPE/POPG lipid bilayers rather than in the aquous subphase. In light of these findings, we may summarize that under equilibrium circumstances, a larger fraction of the total protegrin molecules population will be found in dimeric, or perhaps, oligometic structures on the surface of POPE/POPG lipid bilayers than in bulk water. Certainly, it has been found in [16] that protegrin monomers prefer a transmembrane orientation than one lying flat on the membrane surface. At equilibrium then, it is likely that the majority of protegrin molecules are embedded inside the membrane, in monomeric, dimeric, or even, oligomeric forms, all structural precursors of the biologically relevant pores. These findings notwithstanding, it is important to note that the concept of equilibrium is ill-defined in biological systems. A bacterial membrane may, for example, collapse under the influence of large numbers of antimicrobial peptides. This may occur in time scales comparable to time scales of protegrin self-association and membrane binding, likely rendering kinetics as important as thermodynamics. Indeed a complete mechanistic understanding of how protegrin molecules function may only be plausible by combining both kinetics and thermodynamics studies. This is admittedly beyond the scope of this manuscript.

In order to calculate the peptide-peptide binding affinity we need to define the binding geometry parameters, *D_min_* and *D_max_*. Thus, the minimum separation distance between two peptides is chosen as *D_min_* = 2a = 9 *Å*. When analyzing the restraint forces *F^res^*(*z*) for all three systems, we find that forces *F*(*z*) decrease monotonically with peptide-peptide distance. Especially the PMF on *D_max_* = 23 *Å* calculated using (1) is about 1 *k_B_T*, and for *D_max_* = 25 *Å* it is less than 1 *k_B_T* for all systems. We can suggest that above 25 *Å* we will have sufficient plateaus for our PMF. Therefore the peptide is considered to be bound within *D_max_* = 25 *Å*. Accordingly, using (3), we find a free energy of Δ*G*^0^ = −16.2 ± 0.9 kcal/mol for the formation of a PG1*_p_^d^* dimer in a 150 mM NaCl solution at 310 K. The binding free energy for a PG1*_a_^d^* in water is calculated to be Δ*G*^0^ = −3.5 ± 0.9 kcal/mol. For peptides on the POPE:POPG membrane surface, we calculate a binding free energy for a PG1*_p_^d^* of Δ*G*^0^ = −19.1 ± 1.2 kcal/mol and a binding free energy for a PG1*_a_^d^* of Δ*G*^0^ = −21.8 ± 1.2 kcal/mol. Lastly, for two peptides inserted inside the POPE/POPG membrane the PG1*_p_^d^* binding free energy is Δ*G*^0^ = −7.4 ± 1.3 kcal/mol and the PG1*_a_^d^* one is Δ*G*^0^ = −2.9 ± 1.3 kcal/mol.

### 3.2. The Role of Ionic Bridge and Hydrogen Bond Networks in Dimerization Stability

It is worthwhile stressing that although protegrin molecules carry a large positive charge, they still dimerize strongly. Certainly, the environment somehow mitigates the electrostatic repulsion. Arginines aside, the sequence and structure of the peptides clearly promote stong association. In this section, we analyze molecular dynamics trajectories to elucidate the mechanism of interaction between two PG1 peptides. We focus on the thermodynamically most stable structure, which according to the calculated PMFs has the two peptides at a distance of 11 *Å* apart.

#### Ionic Bridges

**Figure 4:**
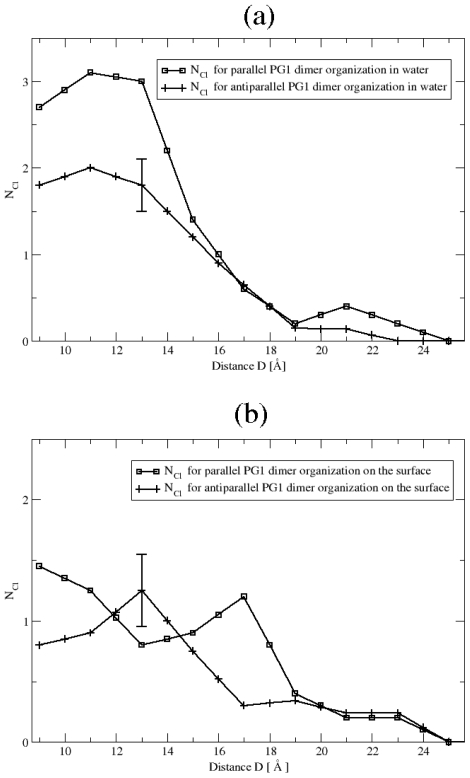
Average number, *N_Cl_* of chloride counterions bound to PG1 peptides as a function of the distance D, between the two peptides, for the parallel and the antiparallel *β*-sheet arrangement **(a)** in water and **(b)** on the surface of the POPE/POPG membrane.

**Figure 5:**
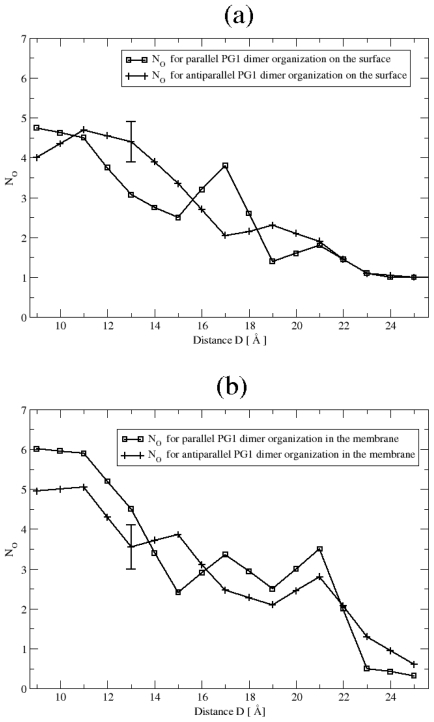
Average number *N_O_* of anionic lipid headgroup oxygens binding to both PG1 peptides as a function of the distance D, between the two peptides in the parallel and the antiparallel *β*-sheet arrangements **(a)** on the surface of the POPE/POPG membrane and **(b)** inside the POPE/POPG membrane.

First, we investigate the formation of ionic bridges between peptides. These are formed when a negatively charged group is proximal to and interacts with argninine residues. We define two distinct types of ionic bridges between two pairs of molecules: the contact solute pairs (CSP) and the solvent-separated solute-peptide pairs (SSSP). CSP bridges form when a negatively charged group, such as a chloride ion or a lipid phosphate group, are in direct and stable contact with argninine residues. SSSP bridges form when there are water molecules (typically one) in-between an arginine and a negative atom, or group. For POPG lipids, there are four types of relevant oxygens: (1) OH oxygens, (2) phosphate oxygens, (3) ester oxygens, and (4) carbonyl oxygens. We examine and average over all four types. In particular, we count a CSP ionic bridge when any arginine guanidinium group (RNHC(NH2)2+) of an arginine residue is found within 4.3 *Å* of a Cl^−^ ion or of any of the anionic lipid head group oxygens. An SSSP ionic bridge is identified when the peptide guanidiniums are within 7.6 *Å* of a Cl^−^ ion or of any of the head group oxygens. More precisely, an ionic bridge is accounted for when these interactions are stably present for at least 10 consecutive simulation picoseconds. Notably, characteristic distance for ionic bridge to be significantly greater than the characteristic distance for hydrogen bonds. That is why we can make suggestion that in different environments we may have different geometrical structure for PG1 dimers. In Figure 4 we present the number of counterions *N_Cl_* bound to both peptides as a function of the distance in Environments 1 and 2. The number of these ionic bridges changes with the distance between the peptides. For long distances between the peptides, more ionic bridges are formed as they come closer to one another. A maximum number of guanidinium-chloride bridges is reached in water when the peptides are approximately 11 *Å* apart, regardless of their orientation, parallel or antiparallel. This is the distance when the PMF reaches its minimum in water for both PG1*_p_^d^* and PG1*_a_^d^*. Notably, the actual number of ionic bridges is higher for the parallel structure than the antiparallel, when the PMF well is deeper for the parallel than the antiparallel comformation. This result is suggestive of the importance of chloride ionic bridges in dimerization of PG1 in water and may explain the preferential dimerization in the parallel comformation. On the membrane surface, the role of chloride bridges in PG1 dimerization ceases to be as important. In Figure 4 we again present the number of chloride ion bridges as a function of the distance between the two peptides, whereas in Figure 5 we present the number of ionic bridges with lipid oxygens, as a function of the distance between the two peptides. Expectedly, when positively charged peptides approach the membrane surface, there is a re-arrangement of ionic bonds, especially at a distance close to the Debye-Huckel screening length [15]. In our case, the corresponding Debye-Huckel length is approximately 10 *Å*. Negatively charged ions are expelled from this area and what we observe is that ionic bridges are now formed between arginines and lipid headgroup oxygens. As a result the numbers of chloride-guanidinium ionic bridges is not as high for peptides on the membrane surface, as it is for peptides in water. Importantly, there is no discernible trend for the number of chloride ionic bridges as a function of the distance. On the other hand, the number of ionic bridges increases as the peptides get closer and reaches a maximum again at a distance of approximately *D* = 11 *Å*, when the PMF is minimum for peptide dimerization on the membrane. Interestingly, the number of ionic bridges the peptides form with lipid oxygens on the membrane surface is approximately identical for the two comformations. Indeed, the PMFs of the parallel and antiparallel structures are not significantly different at their minimum. These results are suggestive of the importance of ionic bridges between peptide arginines and lipid oxygens in the dimerization of PG1 on the surface of lipid bilayers. In Figure 5, we also present the number of ionic bridges the peptides form with lipid oxygens, when they are embedded in the hydrophobic core of the membrane. There is again an increase in the number of ionic bridges with a decreasing peptide-peptide distance. There is however, no strong correlation between the distances where the maximum number of ionic bridges forms and where the PMFs become minimum. The PMFs are of smaller depth and are flatter for peptides inside the membrane than in the other two environments. The absence of trend suggests that ionic bridges are not the determining interaction for dimerization of peptides inside the membrane. Indeed, dimerization in the membrane is expected to be largely dictated by hydrophobic effects.

**Figure 6:**
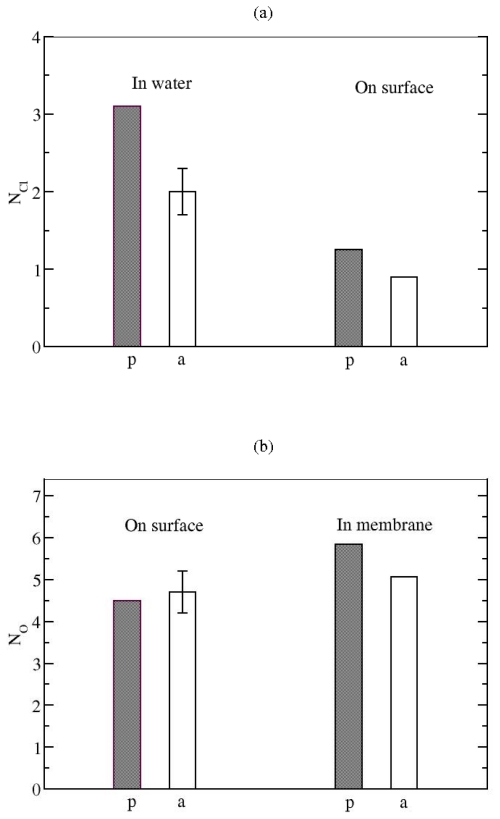
**(a)** Average number, *N_Cl_*, of chloride counterions bound the both PG1 peptides. p: the parallel; a: the antiparallel *β*-sheet arrangement of the PG1 dimer. **(b)** Average number, *N_O_*, of anionic lipid headgroup oxygens bound to the both PG1 peptides, on the surface of the POPE/POPG membrane and inserted in the POPE/POPG membrane. p: the parallel; a: the antiparallel *β*-sheet arrangement of the PG1 dimer.

We can analyze the importance of ionic bridges further by looking at their types more closely. In Figure 6 we present the average number of both chloride and oxygen ionic bridges when the peptides are at a distance *D* = 11 *Å* away. In water, only the chloride ionic bridges are important. Their average number is *N_Cl_* = 3.1 ± 0.3 for the parallel dimer structure, as opposed to *N_Cl_* = 2.0 ± 0.3 for the antiparallel structure. These numbers are averages over the last 4 ns of simulations. Again, according to calculated PMFs (Figure 6), peptides preferrentially dimerize in parallel structures in water. Simulation results suggest that ionic bridges may contribute to the parallel structure being more favorable. On the membrane surface, the number of dimer-bound counterions is equal to *N_Cl_* = 1.2 and *N_Cl_* = 0.9 for parallel and antiparallel structures, respectively. On average, we find that 70% of bound Cl^−^ counterions are in the SSSP states and only 30% in the CSP states for Environments 1 and 2. For peptides on the surface of the membrane, the average numbers, *N_O_*, of anionic lipid headgroup oxygens binding to the PG1 dimer peptides are approximately equal for the parallel and antiparallel arrangements: *N_O_* = 4.5 ± 0.5 and *N_O_* = 4.7 ± 0.5, respectively. We find that almost all ionic bridges (more than 80%) form with the phosphate oxygens. For peptides embedded in the hydrophobic core of the lipid bilayer, the average number of guanidinium-oxygen ionic bridges is *N_O_* = 5.9 ± 0.5 for the parallel dimer and *N_O_* = 5.1 ± 0.5 for the antiparallel one.

#### Hydrogen bonds

**Figure 7:**
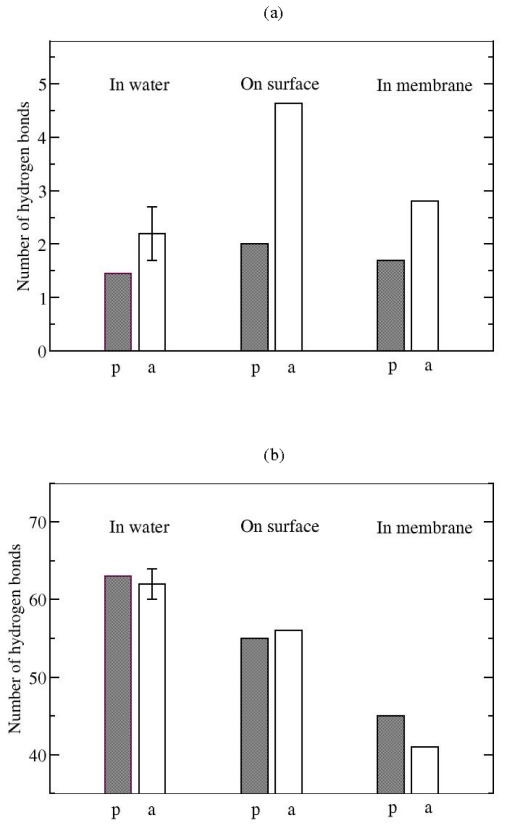
Average number of endogenic **(a)** and exogenic **(b)** hydrogen bonds between two PG1 peptides in all three studied environments. p: the parallel; a: the antiparallel *β*-sheet arrangement of the PG1 dimer.

Next, we analyze the hydrogen bonds between PG1 peptides and between the dimers and their environments. We distinguish these two categories as endogenic, or intramolecular, and exogenic, or external, hydrogen bonds. We identify a hydrogen bond when two candidate atoms are closer than 2.4 *Å* [35]. We present the number of hydrogen bonds averaged over the last 4 ns of simulations. Notably, the PG1 dimer topology diagram [36] suggests six possible intramolecular hydrogen bonds for the parallel and antiparallel orientations (Figure 7). The calculated numbers vary, with the antiparallel orientation having a larger number than the parallel orientation, regardless of the environment. There are more endogenic hydrogen bonds for both orientations on the membrane surface than both in bulk water and inside the membrane. For the dimer on the surface, an average of 2.0 hydrogen bonds were observed for the PG1*_p_^d^* structure and 4.6 hydrogen bonds for the PG1*_a_^d^* structure. On the other hand, for the dimer in the water, only 1.4 and 2.2 hydrogen bonds were found for PG1*_p_^d^* and PG1*_a_^d^*, respectively. A PG1 dimer inserted perpendicularly into the membrane has 1.7 hydrogen bonds in the PG1*_p_^d^* structure and 2.8 in the PG1*_a_^d^* configuration. Calculated PMFs indicate that the PG1 dimer is more stable near the membrane surface in the PG1*_a_^d^* conformation, and the numbers of hydrogen bonds may only to a small extent explain this. On the other hand, taking into account that the relative strength of hydrogen bonds is much weaker than ionic bonds [37], we can assume that the parallel configuration in water is rendered more stable primarily by counterions.

The number of exogenic hydrogen bonds is shown in Figure 7. The numbers are practically identical for PG1*_p_^d^* and PG1*_a_^d^* dimers in bulk water: *N_H_* = 63 ± 2 for the parallel structure and *N_H_* = 62 ± 2 for the antiparallel. The average numbers of exogenous hydrogen bonds are also very similar for the peptide inserted into the membrane: for the parallel orientation the number is *N_H_* = 45, whereas for the antiparallel it is *N_H_* = 41. Overall, no clear discernible trends are observed that are in accord with the PMF calculations. In general, it appears that the number of hydrogen bonds is not a strong determinant of most stable dimer structures, in constast to ionic bridges which appear to better explain the preferential formation of one type of dimer over the other.

## 4. Conclusions

We present free energy calculations of dimerization of the cationic *β*-sheet antimicrobial peptide PG1 in parallel and antiparallel structures in different environments. Our simulation results provide important evidence that the driving force for the dimerization of this peptide inside and outside the membrane is determined by the formation of ionic bridges, more so than the formation of hydrogen bonds. Ionic bridges between peptide arginines and chloride ions dictate the dimerization of PG1 in water, stabilizing the parallel dimer structure. On the surface, dimerization is influenced less by chloride ionic bridges and more by lipid oxygen ones. Inside the hydrophobic core, ionic bridges are no longer the determining interaction, with hydrophobic effects likely dominating.

This work also provides supporting evidence for the existence of the antiparallel state of a PG-1 dimer on the surface of the membrane. Although the parallel orientation is dominant in water and in the transmembrane inserted state, the antiparallel competes with the parallel one on the surface of the lipid membrane. The results of PMF simulations are consistent with the results of NMR experimental observations which determined that the PG1 dimer adopts an antiparallel structure upon binding to DPC micelles [7] and a parallel NCCN packing structure in a transmembrane orientation inside POPE/POPG membranes [3].

Calculation of PMFs for PG1 dimers and their complexes, particularly inside the membrane, may be useful in elucidating the pathway of action and selectivity of this peptide. Importantly, these calculations may help explain biological functions of antimicrobial peptides in terms of biophysical interactions.
